# Spatial heterogeneity and temporal dynamics of mosquito population density and community structure in Hainan Island, China

**DOI:** 10.1186/s13071-020-04326-5

**Published:** 2020-09-04

**Authors:** Yiji Li, Guofa Zhou, Saifeng Zhong, Xiaoming Wang, Daibin Zhong, Elizabeth Hemming-Schroeder, Guohui Yi, Fengyang Fu, Faxing Fu, Liwang Cui, Guzhen Cui, Guiyun Yan

**Affiliations:** 1 Department of Pathogen Biology, Hainan Medical University, Haikou, Hainan China; 2 Program in Public Health, College of Health Sciences, University of California, Irvine, CA 92697 USA; 3 Public Research Laboratory, Hainan Medical University, Haikou, Hainan China; 4 Department of Medical Technology, Chongqing Medical and Pharmaceutical College, Chongqing, China; 5 Department of Internal Medicine, Morsani College of Medicine, University of South Florida, Tampa, FL 33612 USA; 6 Key Laboratory of Medical Microbiology and Parasitology of Education Department of Guizhou, School of Basic Medical Science, Guizhou Medical University, Guiyang, China; 7 Key Laboratory of Endemic and Ethnic Diseases Ministry of Education, Guiyang, China

**Keywords:** Hainan Island, Mosquito composition, Population dynamics, Species diversity, BGS trap, CDC light trap

## Abstract

**Background:**

Mosquitoes are vectors of many tropical diseases. Understanding the ecology of local mosquito vectors, such as species composition, distributions, population dynamics, and species diversity is important for designing the optimal strategy to control the mosquito-borne diseases.

**Methods:**

Entomological surveillance of adult mosquitoes was conducted in five sites representing different ecological settings across Hainan Island from January to December of 2018 using BG Sentinel (BGS) traps and Centers for Disease Prevention and Control (CDC) light traps. In each site, we selected three areas representing urban, suburban and rural settings. Eighteen trap-days were sampled in each setting at each site, and CDC light traps and BGS traps were setup simultaneously. Mosquito species composition, distribution, population dynamics, and species diversity were analyzed. Mosquito densities were compared between different study sites and between different settings.

**Results:**

Nine species of mosquitoes belonging to four genera were identified. *Culex quinquefasciatus* (80.8%), *Armigeres subalbatus* (13.0%) and *Anopheles sinensis* (3.1%) were the top three species collected by CDC light traps; *Cx. quinquefasciatus* (91.9%), *Ae. albopictus* (5.1%), and *Ar. subalbatus* (2.8%) were the top three species collected by BGS traps. Predominant species varied among study sites. The population dynamics of *Ae. albopictus*, *An. sinensis* and *Cx. quinquefasciatus* showed clear seasonal variation regardless of study sites with a varied peak season for different species. Mosquito abundance of all species showed significant differences among different study sites and among urban, suburban and rural areas. Danzhou had the highest mosquito biodiversity, with an α, β, and Gini-Simpson biodiversity index of 8, 1.13 and 0.42, respectively. BGS traps captured *Aedes* mosquito at a higher efficiency than CDC light traps, whereas CDC light traps captured significantly more *Anopheles* and *Armigeres* mosquitoes than BGS traps.

**Conclusions:**

Mosquitoes were abundant on Hainan Island with clear seasonality and spatial heterogeneity. Population density, species composition, distribution, and species diversity were strongly affected by the natural environment. Different tools are required for the surveillance of different mosquito species.
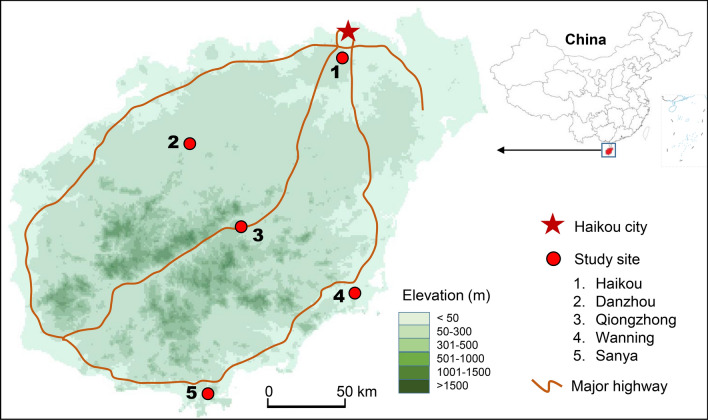

## Background

Mosquitoes transmit many diseases, such as malaria, filariasis, dengue fever, chikungunya fever, Japanese encephalitis, and yellow fever [1, 2]. Vector control is an essential component of public health interventions to reduce the transmission of these diseases [3, 4]. Furthermore, mosquito surveillance has become increasingly important due to the continued global threat of malaria, dengue virus (DENV), Zika virus (ZIKV), chikungunya virus (CHIKV), and West Nile virus (WNV). Knowledge of mosquito population dynamics, species distribution, and species diversity is essential for developing cost-effective vector surveillance tools [5]. Many factors affect mosquito population dynamics and species distribution, including climatic conditions [6], urbanization [7], and local ecological settings [8]. Mosquito population diversity and seasonal fluctuations can in turn affect the risk of mosquito-borne diseases [9]. Therefore, studies of mosquito species diversity and population dynamics can help to develop better management strategies for mosquito-borne diseases [10, 11]. Appropriate surveillance tools for efficient mosquito population monitoring is a critical step in developing and implementing appropriate strategies to control vector populations.

Historically, mosquito-borne infectious diseases, such as malaria, dengue fever, and filariasis, have been prevalent on Hainan Island, China. For example, Hainan Island had a very severe malaria epidemic in the 1950s and island-wide dengue fever pandemics in late 1970s and early 1990s [12–16]. *Anopheles sinensis*, *Anopheles dirus*, *Anopheles vagus*, *Anopheles maculipalpis*, *Anopheles tessellatus* and *Anopheles minimus* were the major malaria vectors in the past [17–19]. The 1979 and 1991 dengue fever outbreaks in Hainan, which are believed to be caused mainly by *Aedes aegypti* [16, 19], led to a total of 604,854 cases and 475 deaths. The most recent local dengue outbreak in Hainan occurred in 2019 [20], indicating the reemergence of the virus on the island after its cessation in 1991. While *Aedes aegypti* was the major dengue vector in the 1970s and 1990s [19], recent studies have shown a trend of increasing *Aedes albopictus* abundance and decreasing *Ae. aegypti* abundance since then [21, 22]. Filariasis is also one of the historical epidemics of mosquito-borne infectious disease in Hainan till the 1980s [23]. Japanese encephalitis (JE) was used to be a major mosquito-borne infectious disease all over China including Hainan in the past, and *Culex* is the major vector [24, 25]. Due to the available and widely implemented vaccination, JE has been suppressed substantially in China [24]; however, it remains a public health threat all over South, East and Southeast Asia [26]. The recent reemergence of mosquito-borne diseases, such as dengue fever, has become a new threat to public health on Hainan Island [20]. Nearly 40 species of *Anopheles* mosquitoes have been reported on Hainan Island in the past [19]. The dengue fever vector *Ae. aegypti* has also been reported [19], and *Ae. albopictus*, another vector of dengue fever, is widely distributed [27]. More than 18 species of *Culex* mosquitoes have been previously reported from Hainan Island [19]. However, no systematic mosquito surveillance has been conducted recently. Continuous monitoring of mosquito populations remains a key focus of mosquito-borne infectious disease prevention and control.

Thus, the aim of this study was to determine the mosquito population dynamics and species diversity in different ecological settings on Hainan Island. We used BGS and CDC light traps simultaneously for mosquito sampling to examine the differences in mosquito captures between the two tools and assess whether different tools are needed for mosquito population surveillance.

## Methods

### Study areas

Hainan Island located in the tropical area of South China Sea, with an area of 33,920 km^2^ and a population of 8.6 million [17]. The annual average temperature is 21.6 °C, and annual cumulative precipitation is 1980 mm. This climate is ideal for the development and reproduction of most mosquito species [28].

**Fig. 1:**
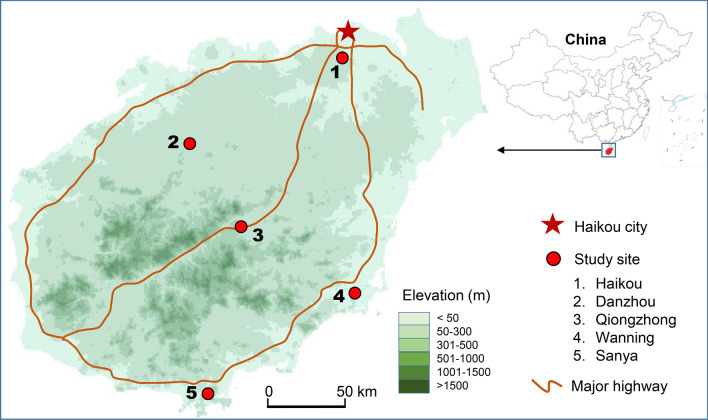
Map of the study sites in Hainan Island, China

Field sampling was conducted from January to December 2018 in five sites on Hainan Island, in Hainan Province, China (Fig. 1). We selected the following five locations in different geographical areas for mosquito surveillance: Haikou City, the capital of Hainan Province and the largest city in the island province; Sanya City, a major tourist destination; Wanning City; Danzhou City; and Qiongzhong City, the only mountainous site in the central area of the island (Table 1). The five sites belong to three climate zones; annual precipitation varies a lot from 1315 mm in Sanya to 2230 mm in Qiongzhong (Table 1). In addition, although there is only one rainy season in all sites, the rainy season lasts from May to October in Haikou, Sanya and Danzhou, from April to November in Qiongzhong and April to December in Wanning.

**Table 1:** Description of study sites in Hainan Island, China

Study site	Location	Elevation (m.a.s.l.)	Landscape	Climate zone	Annual mean temperature (°C)	Annual precipitation (mm)
Haikou	110°19’E, 19°59’N	25	Highly urbanized	Humid subtropical	24.2	1650
Danzhou	109°35’E, 19°30’N	46	Plain, agriculture	Tropical humid monsoon	23.8	1830
Qiongzhong	109°50’E, 19°02’N	213	Mountain, forest	Tropical marine monsoon	23.1	2230
Wanning	110°22’E, 18°48’N	10	Mountain and plain	Tropical savanna	25.0	2040
Sanya	109°32’E, 18°19’N	8	Major tourist place	Tropical savanna	25.9	1315

*Abbreviation*: m.a.s.l., meters above sea level

### Surveillance methods and sampling design

Two types of mosquito traps were used: CDC light traps (Lucky Star Environmental Protection Technology Co., Ltd., Wuhan, China), as well as BG Sentinel trap (BioGents, Regensbourg, Germany) with BG-Lure (BioGents, GmbH, Regensbourg, Germany).

We selected an urban, suburban, and rural area in each site for mosquito collection. The selection of the three areas at each site was to represent different ecological settings in each site and minimize differences in ecological effects. Nine traps of each of the two trap types were placed in the five study sites (Fig. 1), and sampling was carried out three to six consecutive days each month in each site. The distance between two traps was at least 40 m. Traps were placed in randomly selected residential areas. The adult mosquito populations were monitored continuously from January 2018 to December 2018. CDC light traps were hung in trees 0.8 m above the ground, whereas BGS traps were placed on the ground. Both traps were setup in the evening, and traps were collected after 24 h. Every 24 h was counted as one trapping period. Mosquitoes were collected and transported to the laboratory for species identification [29]. The geographical coordinates of each study site were recorded using eTrex H portable global positioning system (GPS) devices (Garmin eTrex H).

### Statistical analysis

Mosquito density and comparison of differences were based on square-root transformed number of captures, as this transformation ensured overall data normality. Mosquito density was calculated as the number of mosquitoes per trap per trapping period, adults/trap-day.

For species diversity analyses, we calculated α, β and γ diversity, as well as the Gini-Simpson diversity index based on raw records [30, 31]. α-diversity refers to the average species diversity in a habitat or specific area, which is a local measure of diversity. β-diversity refers to the ratio between local or α-diversity and regional diversity, i.e. the diversity of species between two habitats or regions. γ-diversity is the total diversity of a landscape and is a combination of α and β diversity. True β diversity is the Whittaker’s original definitions of β-diversity [30, 31]. Absolute species turnover quantifies how much more species diversity the entire dataset contains than an average subunit within the dataset. This can also be interpreted as the total amount of species turnover among the subunits in the dataset [32]. The Gini-Simpson index accounts for how individuals are distributed among species in different study sites [33]. The formula for the Gini-Simpson index is $G = 1 - \mathop \sum \limits_{i = 1,n} p_{i}^{2}$, where $p_{i}$ is the proportion of species *i* in a given study site.

Differences in monthly average of mosquito abundance (time series) among different study sites were tested using one-way analysis of variance (ANOVA) with repeated measures after data transformation. Pairwise differences in mosquito abundance were determined using the ANOVA Tukey-Kramer HSD *post-hoc* test with a significance level of 0.05. Statistical analysis was carried out using JMP 9.0 statistical software (JMP, SAS Institute Inc., Cary, NC, USA).

A generalized linear mixed model (GLMM) with Poisson error and log link function was used to analyze the effects of trap configurations and study sites on the numbers of mosquitoes collected based on raw numbers [34]. The data used for GLMM analysis was pooled data of all mosquito species for each genus. To test for study site effects, sampling months were used as covariates. To compare differences in trapping methods, both study sites and sampling months were used as covariates. In addition, a temporal correlation effect was modelled using AR (1) correlation structure. GLMM analysis was performed using R 3.6.3.

## Results

### Mosquito population composition

A total of 1505 and 1337 trap-days of CDC light trap and BGS trap were conducted during the survey period. Using CDC light traps, we collected a total of 44,778 mosquitoes belonging to four genera and at least nine species (Table 2). Among the CDC light trap catches, 36,159 were *Cx. quinquefasciatus*, 5835 *Armigeres subalbatus*, 1380 *An. sinensis*, 696 *Ae. albopictus*, 449 *Cx. tritaeniorhynchus*, and others (*An. vagus*, *Cx. bitaeniorhynchus*, *An. aconitus* and *An. hyrcanus* group) (Table 2). *Culex quinquefasciatus* (80.8%), *Armigeres subalbatus* (13.0%) and *An. sinensis* (3.1%) were the top three species collected by CDC light traps. Using BGS traps, we collected a total of 43,387 mosquitoes belonging to four genera and at least five species (Table 1). Among them, 39,889 were *Cx. quinquefasciatus*, 2208 *Ae. albopictus*, 1214 *Ar. subalbatus*, 70 *An. sinensis*, and others (*Cx*. *tritaeniorhynchus* and *Cx*. *bitaeniorhynchus*) (Table 2). *Culex quinquefasciatus* (91.9%), *Ae. albopictus* (5.1%) and *Ar. subalbatus* (2.8%) were the top three species collected by BGS traps. No *Ae. aegypti* were collected during the study period.

**Table 2:** Descriptive summary of trap data by mosquito species

Species	CDC light trap	BGS trap
Number of trap days	1505	1337
*Anopheles sinensis*	1380 (3.1)	70 (0.2)
*Anopheles vagus*	89 (0.2)	0
*Anopheles aconitus*	4 (<0.1)	0
*Anopheles hyrcanus* group	3 (<0.1)	0
*Aedes albopictus*	696 (1.6)	2208 (5.1)
*Culex quinquefasciatus*	36159 (80.8)	39889 (91.9)
*Culex tritaeniorhynchus*	449 (1.0)	6 (<0.1)
*Culex bitaeniorhynchus*	163 (0.4)	0
*Armigeres subalbatus*	5835 (13.0)	1214 (2.8)
Total number	44778 (100)	43387 (100)

*Notes*: Data represent total number of mosquitoes (percentage of total)

For CDC light traps, among all study sites, Danzhou had the highest density of *Culex* (52.55 ± 10.13 adults/trap-day), *Anopheles* (4.37 ± 0.84 adults/trap-day) and *Armigeres* (14.53 ± 2.57 adults/trap-day) mosquitoes (Tukey-Kramer HSD all *P* < 0.05) (Table 3). Danzhou and Sanya had the highest density of *Aedes* mosquitoes, with 0.76 ± 0.11 and 0.76 ± 0.15 adults/trap-days, respectively (Tukey-Kramer HSD both *P* < 0.05) (Table 3). For BGS traps, Haikou had the highest *Aedes* mosquito density (2.51 ± 0.43 adults/trap-day) (Tukey-Kramer HSD *P* < 0.05); Wanning had the highest density of *Culex* mosquitoes (49.08 ± 9.18 adults/trap-day) (Tukey-Kramer HSD *P* < 0.05); and Danzhou had the highest density of *Anopheles* (0.26 ± 0.08 adults/trap-day) and *Armigeres* (3.70 ± 0.57 adults/trap-day) mosquitoes (Tukey-Kramer HSD both *P* < 0.05) (Table 3).

**Table 3:** Mean mosquito density by study site and mosquito genus

Site	*n*	*Aedes*	*Culex*	*Anopheles*	*Armigeres*
BGS trap					
Haikou	297	2.51 ± 0.43	25.73 ± 2.48	0	0.14 ± 0.03
Danzhou	209	1.13 ± 0.14	25.54 ± 2.81	0.26 ± 0.08	3.70 ± 0.57
Qiongzhong	179	0.39 ± 0.07	14.15 ± 1.78	0.04 ± 0.02	0.58 ± 0.11
Sanya	240	1.64 ± 0.22	17.34 ± 2.32	0.03 ± 0.01	0.56 ± 0.10
Wanning	412	1.85 ± 0.20	49.08 ± 9.18	0	0.07 ± 0.02
CDC light trap					
Haikou	323	0.43 ± 0.09	9.85 ± 0.85	0.08 ± 0.02	0.49 ± 0.09
Danzhou	260	0.76 ± 0.11	52.55 ± 10.13	4.37 ± 0.84	14.53 ± 2.57
Qiongzhong	234	0.26 ± 0.05	14.37 ± 1.89	0.75 ± 0.14	2.86 ± 0.37
Sanya	275	0.76 ± 0.15	7.32 ± 0.50	0.24 ± 0.04	3.09 ± 0.41
Wanning	413	0.20 ± 0.02	35.26 ± 3.47	0.17 ± 0.05	0.83 ± 0.10

*Abbreviation*: *n*, number of collections

### Population dynamics of the predominant species

**Fig. 2:**
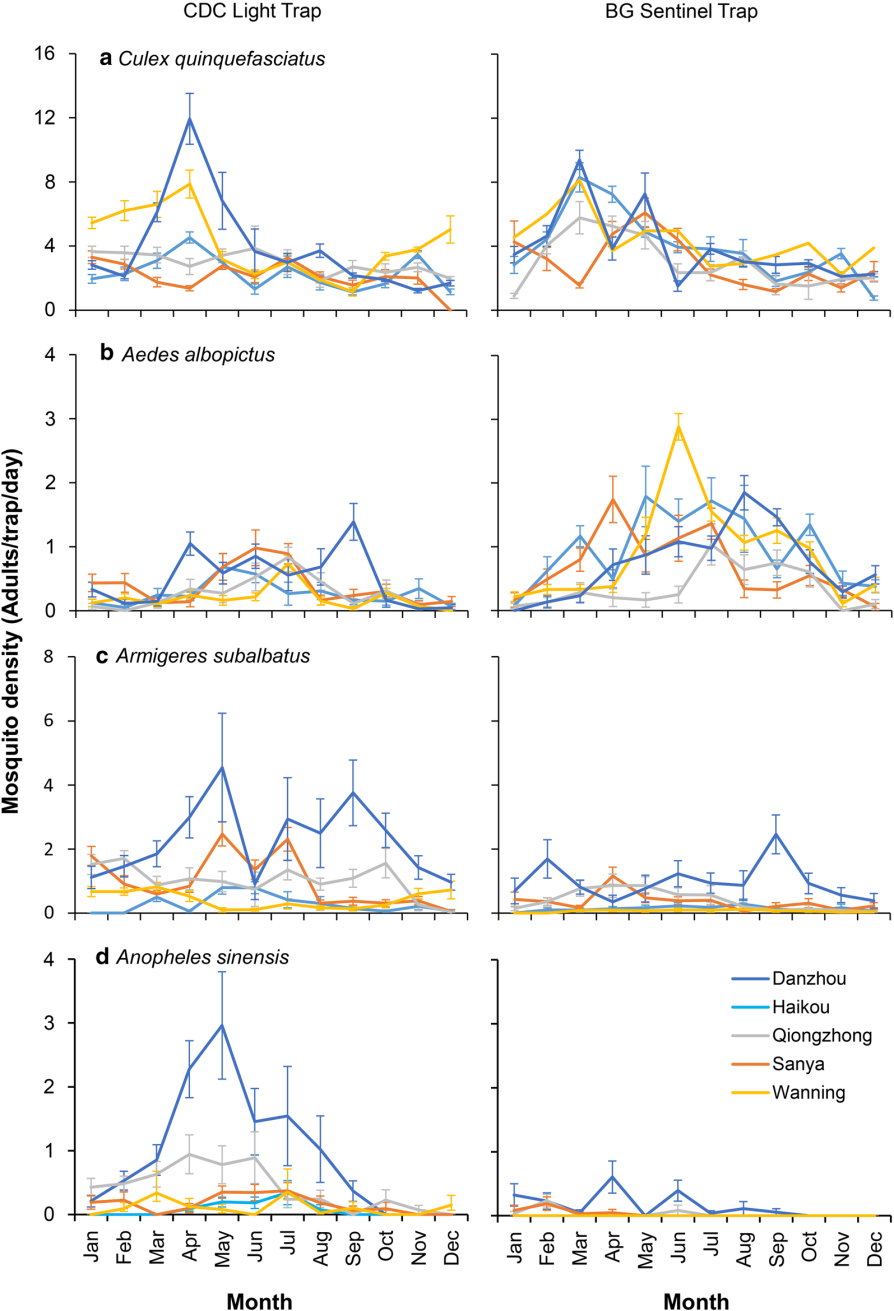
Dynamics of major mosquito species in Hainan Island, China 2018. **a** *Culex quinquefasciatus*. **b** *Aedes albopictus*. **c** *Armigeres subalbatus*. **d** *Anopheles sinensis*. The left panels show mosquito captures by CDC light trap, the right panels show mosquito captures by BG Sentinel Trap. Mosquito abundance is square-root transformed, and values are the mean ± standard error

Here we focused on four major species, i.e. *Cx. quinquefasciatus*, *Ae. albopictus*, *Ar. subalbatus* and *An. sinensis.* Figure 2 shows the population dynamics of these four species at our study sites by different trapping methods. The population dynamics of *Cx. quinquefasciatus* showed clear seasonal variation regardless of study sites and trapping methods, although some sites showed a low density overall (Fig. 2a). The peak months were in general from March to May (Fig. 2a). The population dynamics of *Ae. albopictus* captured by BGS traps showed significant seasonal changes, with a peak collection likely between March and October of the year. CDC light trap collections showed significantly lower density overall, as compared to BGS traps, but CDC light traps revealed a slight peak of *Ae. albopictus* from April to July (Fig. 2b). For both *Ar. subalbatus* and *An. sinensis*, BGS was not an effective surveillance tool (with very few adults captured) and showed minimal seasonal variation in comparison to CDC light traps (Fig. 2c, d). For both species, CDC light trap catches showed a strong seasonality with peak collection from April to September for *Ar. subalbatus* and from April to August for *An. sinensis* (Fig. 2c, d).

### Mosquito species diversity

For this analysis, data were pooled for each study site. In total, nine species (genera) were collected (Table 4). At the species level, Haikou had the lowest α diversity (6 species) and lowest Gini-Simpson diversity index (0.17), while Danzhou had the highest α diversity (8 species) and Gini-Simpson diversity index (0.42). Species turnover was low (from 0 to 2) for all paired-sites (Table 4). Overall, in consideration of the number of individuals captured, species diversity was generally low, as the average Gini-Simpson index was 0.14 and ranged from 0.07 in Wanning to 0.42 in Danzhou (Table 4).

**Table 4:** Species and individual level diversity analysis by location

Study areas	Haikou	Qiongzhong	Danzhou	Wanning	Sanya	Total
Species level						
γ diversity (overall)						9
α diversity (species richness)	6	8	8	7	7	9
True β diversity	1.50	1.13	1.13	1.29	1.29	–
Proportional species turnover β_p_	0.33	0.11	0.11	0.22	0.22	–
Individual level						
Gini-Simpson biodiversity index	0.17	0.31	0.42	0.07	0.38	0.14

*Notes*: Diversity measure types: α denotes the diversity within a study site, higher values mean higher diversity; β denotes differences in diversity between samples from a study site and the overall, a value of 1 means that the study site has all the species found in all study sites; γ denotes the total diversity over the set of all samples; β_p_ represents the proportional difference between overall diversity and the diversity in specific study site

### The impact of study areas, urbanized setting, and trapping methods on mosquito abundance

Overall, all *Aedes*, *Culex*, *Anopheles* and *Armigeres* population densities were significantly different among trapping methods and study sites (all *P* < 0.001, Table 5). Poisson regression analyses showed that CDC light traps had a lower efficiency than BGS traps in capturing *Aedes* and *Culex* mosquitoes (*Z* = − 29.9, *P* < 0.001 for *Aedes*; *Z* = − 32.4, *P* < 0.001 for *Culex*). However, CDC light traps had a higher efficiency in capturing *Anopheles* and *Armigeres* mosquitoes (*Z* = 20.9, *P* < 0.001 for *Anopheles*; *Z* = 40.4, *P* < 0.001 for *Armigeres*).

**Table 5:** Poisson regression analysis of differences in mosquito density for each genus. Intercept terms are not shown

Genus		*df*	*χ*^2^	*P*
*Aedes*	Month	1	0.96	0.3268
	Method	1	1031.13	< 0.00001
	Site	4	349.92	< 0.00001
	Area	2	292.31	< 0.00001
	Method*Site	4	241.29	< 0.00001
	Method*Area	2	6.12	0.0470
	Site*Area	8	178.76	< 0.00001
	Method*Site*Area	8	198.67	< 0.00001
*Culex*	Month	1	21955.76	< 0.00001
	Method	1	1076.99	< 0.00001
	Site	4	18709.47	< 0.00001
	Area	2	5157.04	< 0.00001
	Method*Site	4	4894.59	< 0.00001
	Method*Area	2	2298.21	< 0.00001
	Site*Area	8	22579.33	< 0.00001
	Method*Site*Area	8	3164.88	< 0.00001
*Anopheles*	Month	1	333.49	< 0.00001
	Method	1	1370.70	< 0.00001
	Site	4	2810.98	< 0.00001
	Area	2	2312.00	< 0.00001
	Method*Site	2	17.23	0.0002
	Method*Area	1	4.16	0.0413
	Site*Area	7	164.47	< 0.00001
	Method*Site*Area	2	1.76	0.4139
*Armigeres*	Month	1	372.03	< 0.00001
	Method	1	2955.65	< 0.00001
	Site	4	9305.74	< 0.00001
	Area	2	4761.00	< 0.00001
	Method*Site	4	74.95	< 0.00001
	Method*Area	2	727.38	< 0.00001
	Site*Area	7	154.47	< 0.00001
	Method*Site*Area	2	1250.70	< 0.00001

*Notes*: Method: CDC light trap and BGS trap; Site: Haikou, Sanya, Wanning, Danzhou and Qiongzhong; Area: urban, suburban and rural

*Abbreviation*: *df*, degrees of freedom

*Aedes* was the only species that did not show significant differences in densities among months, and *Anopheles* was the only species that did not show significant interactions among trapping methods, study sites and study areas. (Table 5)

**Fig. 3:**
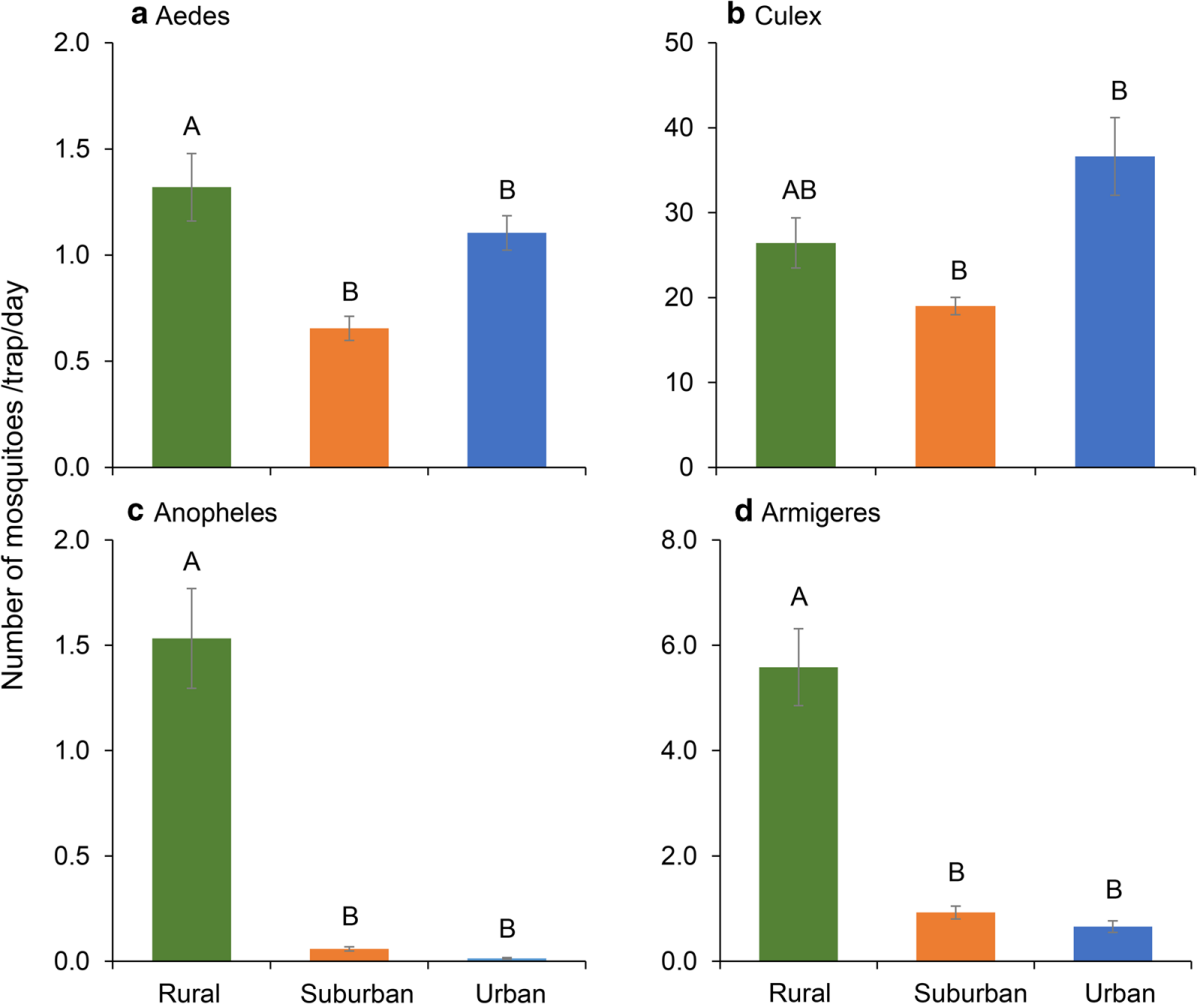
Mosquito densities in different urbanized setting. **a** *Aedes*. **b** *Culex*. **c** *Anopheles*. **d** *Armigeres*. Values are the mean ± standard error. Bars labelled with different letters within the same panel are significantly different from each other (*P* < 0.05)

Data were pooled from different sites for analyzing differences in mosquito abundance among urban, suburban and rural areas. Mean mosquito abundance showed strong differences among urban, suburban and rural areas (Fig. 3). For both *Aedes* and *Culex* mosquitoes, the abundance was lowest in suburban areas, whereas, population abundance for both genera were similar in rural and urban areas (Fig. 3a, b). Over 95% of *Anopheles* mosquitoes were found in rural areas, and about 80% of *Armigeres* were collected from rural areas. Both mosquito genera showed no difference in abundance between urban and suburban areas (Fig. 3c, d). Poisson regression analysis showed significant differences in mosquito abundances among collection areas for all mosquitoes after adjustments for sampling months, sampling methods and study sites (Table 5).

## Discussion

Hainan Island has a tropical climate and complex terrain. It has been reported that there are many mosquito species distributed across the island in the past [21, 35]. In our study, we found that there are at least nine mosquito species present on Hainan Island. *Anopheles*, *Aedes, Culex* and *Armigeres* mosquitoes, which are found on the island, are important vectors of mosquito-borne diseases, such as malaria, dengue, filariasis, zika, chikungunya. *Armigeres subalbatus* is the vector of the zoophilic *Wuchereria bancrofti*, which causes filariasis in humans [36]. In our study, *An. sinensis*, *Cx. quinquefasciatus*, *Ae. albopictus* and *Ar. subalbatus* were found to be widely distributed across the island and occurring at high densities. We also found *Cx. quinquefasciatus*, *Ar. subalbatus* and *Ae. albopictus* year round. The presence of these species throughout the year can be explained by the tropical climate on the island with plenty of year-round rainfall, and thus breeding habitats are widely available and perennial. Malaria, dengue fever and other mosquito-borne tropical diseases were all prevalent on the island in the recent past [15, 20]. Therefore, vector surveillance is important for the control and prevention of mosquito-borne diseases.

We found strong heterogeneity in mosquito population distributions, as mosquito density of all genera, *Anopheles*, *Aedes*, *Culex* and *Armigeres*, varied significantly among the five study sites, which is likely a reflection of differences in ecological settings. Different ecological conditions, urban residential, suburban mixed residential and agricultural, and rural agricultural areas, also impacted the distribution and density of mosquito species, a finding which is consistent with previous studies [37–39]. For example, rural areas had a significantly higher density of *Anopheles* and *Armigeres* mosquitoes than that in urban and suburban areas. Thus, optimal surveillance and mosquito control strategies may be required in different places, so as to adapt to the local mosquito distribution and dynamics in different areas.

Furthermore, *Ae. albopictus* survive in the tropical Hainan Island throughout the year, and the peak season has expanded to April-October in 2018. The peak season in Hainan Island is longer than the average peak season of *Ae. abopictus* in north and central China, which is June-September [40]. The tropical climate of Hainan Island is likely more suitable for the survival of *Ae. abopictus*, and thus Hainan Island may be at a higher risk of future dengue outbreaks. Previous studies in Guangzhou and other places showed that urban areas had a higher proportion of container type habitats, whereas more agricultural related habitats are found in the rural area. These two habitat types support the breeding of different mosquito species [37, 41], which may in part explain the difference in species composition in these areas. However, there is no detailed habitat availability evidence from Hainan Island, so further studies are needed.

There were differences in mosquito catches between CDC light traps and BGS traps. In our study, CDC light traps captured nine species of mosquitoes, whereas BGS traps only captured five species. However, the two methods may be complementary and thus, used in conjunction with each other for population monitoring, as they work differently on certain genera. For example, CDC light traps captured *Anopheles* and *Armigeres* mosquitoes more effectively, while BGS traps captured *Aedes* mosquitoes at a higher rate, which is consistent with previous studies [3, 42, 43]. The two tools work equally well in capturing *Culex* mosquitoes. While CDC light traps captured more species overall, the two methods complement each other by their differential effectiveness at capturing mosquito genera. There are also many other mosquito sampling methods, such as human landing catch (HLC), animal-baited traps, landing boxes, pit shelters, etc. [44, 45]. Each sampling method may be suitable for different mosquito species. For example, zoophilic mosquitoes such as *Anopheles arabiensis* are more attracted to cattle than humans, so a cattle-baited trap is a potentially more effective method for sampling this species than human landing catches [46]. Each trapping method has its advantages and disadvantages, which have been reported in detail by Lima et al. [47] and Killeen et al. [48]. The effectiveness of other sampling methods in our study area require further study.

Interestingly, in our study, we did not find any *Ae. aegypti*. *Aedes aegypti* used to be the dominant *Aedes* species on Hainan Island and was responsible for dengue fever pandemics on the island from 1970s–1990s [19]. *Aedes aegypti* was last reported on the island in 2012 from a few isolated places [21]. *Aedes albopictus* has been reported throughout the island in the past few years [21, 22]. Whether the disappearance of *Ae. aegypti* in Hainan is the result of replacement by *Ae. albopictus* is worthy of further investigation. If there is a species replacement, questions related to how species replacement occurs and how species replacement affects dengue virus transmission require further investigation.

*Armigeres* (*Armigeres*) *subalbatus* is a species complex of zoophilic mosquitoes belonging to the genus *Armigeres*, which is the least studied genus of mosquitoes captured during this study with regard to public health [36, 49, 50]. It is a natural vector for filarial worms of livestock, which causes filariasis in humans [36, 50]. This species has also been found at a low density and carrying virus in Yunnan, China [50]. Moreover, a study in central China found that *Ar. subalbatus* mosquitoes may carry many different viruses [51]. Indeed, *Ar. subalbatus* has been confirmed as a vector of many virus [52]. In this study, we found a high density (compared to *Aedes* and *Anopheles*) of mosquitoes belonging to the *Ar. subalbatus* complex year-round, which may have public health implications given the vector potential of this complex. However, limited by the study aims, we did not test the infection status of all mosquitoes, but this line of inquiry may be worthy of further investigations.

In our study, we only collected nine species of mosquitoes on Hainan Island in 2018, which is a substantially lower number than collected in previous studies in Hainan [53]. One of the reasons for this discrepancy is likely due to the trapping locations. In this study, we mainly placed mosquito traps near residential areas. However, some mosquito species, such as *An. dirus* and *An. minimus* are known to inhabit mountainous areas with forest or forest edges [54]. Both *An. dirus* and *An. minimus* are malaria vectors which were present on the island in the past [28], but they may have been simply missed by our experimental designs. Secondly, Hainan Island is experiencing rapid development, urbanization, road construction and other developments, which may have caused environmental modifications that in turn affect larval habitability and mosquito survival of some species. Many studies have demonstrated that changes in the landscape can affect the distribution of the local mosquito vectors [55–57]. Future sampling should cover more diverse ecological settings, so that results can reflect the true species diversity of the mosquito community.

## Conclusions

We carried a year-long, multi-site mosquito population dynamics study in Hainan, China. The results indicated that BGS traps and CDC light traps complement each other for surveillance of different mosquito vectors. Additionally, mosquito density, population dynamics and diversity showed strong spatial heterogeneity. Therefore, different intervention strategies may be required in different geographical regions and land use types, in order to achieve robust and cost-effective vector surveillance and control for reducing mosquito-borne disease risk.

## Data Availability

All data generated or analysed during this study are included in this published article.

## Abbreviations

DF: dengue fever
ZIKV: Zika virus
CHIKV: chikungunya virus
WNV: West Nile virus
JE: Japanese encephalitis
BGS trap: BG sentinel trap
CDC light trap: Centers for Disease Control and Prevention light trap
